# Adherence to the low carbohydrate diet and the risk of breast Cancer in Iran

**DOI:** 10.1186/s12937-019-0511-x

**Published:** 2019-12-12

**Authors:** Bahareh Sasanfar, Fatemeh Toorang, Ahmad Esmaillzadeh, Kazem Zendehdel

**Affiliations:** 1 Cancer Research Center, Cancer Institute of Iran, Tehran University of Medical Sciences, P.O. Box: 13145158, Tehran, Iran; 2 Department of Community Nutrition, School of Nutritional Sciences and Dietetics, Tehran University of Medical Sciences, Tehran, Iran; 3 Obesity and Eating Habits Research Center, Endocrinology and Metabolism Molecular-Cellular Sciences Institute, Tehran University of Medical Sciences, Tehran, Iran; 4 Department of Community Nutrition, School of Nutrition and Food Science, Isfahan University of Medical Sciences, Isfahan, Iran; 5 Cancer Biology Research Center, Cancer Institute of Iran, Tehran University of Medical Sciences, Tehran, I.R Iran; 6 Breast Diseases Research Center, Cancer Institute of Iran, Tehran University of Medical Sciences, I, Tehran, R Iran

**Keywords:** Diet, Carbohydrate, Fat, Protein, Macronutrient, Breast cancer

## Abstract

**Background:**

Previous studies on the link between macronutrients and breast cancer have mostly focused on individual macronutrients rather than their combination. This study investigates the association between adherence to a low carbohydrate diet and odds of breast cancer among women.

**Methods:**

This hospital-based case-control study was carried out on 412 women with pathologically confirmed breast cancer within the past year and 456 apparently healthy controls that were matched in terms of age and residential place. Dietary data was collected using a 168-item validated FFQ. Participants were classified in terms of quintiles of percentages of energy intake from carbohydrates, proteins, and fats. Then, individuals in the highest quintile of fat and protein intake were given a score of 5 and those in the lowest quintile of these macronutrients were given a score of 1. Participants in the other quintiles of these macronutrients were given the corresponding score. In terms of carbohydrate intake, those in the highest quintile received a score of 1 and those in the lowest quintile received 5. The scores were then summed up to calculate the total low carbohydrate diet (LCD) score, which varied from 3 to 15. A higher score meant greater adherence to a low carbohydrate diet.

**Results:**

The mean age of study participants was 45.2 y and mean BMI was 28.4 kg/m^2^. Mean LCD score of participants was 8.9 ± 2.5 (8.9 ± 2.6 in cases and 9.0 ± 2.5 in controls). Although no significant association was observed between adherence to the LCD score and odds of breast cancer in the study population, a trend toward significant positive association was seen between consumption of LCD and odds of breast cancer in postmenopausal women; after controlling for several potential confounders, individuals in the third quartile of LCD score were 1.94 times more likely to have breast cancer than those in the lowest quartile (95% CI: 1.00, 3.76). This association strengthened after controlling for dietary variables (2.50; 1.18–5.32). Even after further adjustment for BMI, this association remained significant (2.64, 1.23–5.67). No significant relationship was observed in premenopausal women, either before or after controlling for confounders.

**Conclusion:**

Adherence to LCD may be associated with increased odds of breast cancer in postmenopausal women. Prospective cohort studies are needed to confirm these findings.

## Introduction

Low- carbohydrate diets (LCD) are popular diets, and studies have reported beneficial effects on several chronic diseases, including obesity [1], diabetes [2], epilepsy [3, 4], cardiovascular diseases [5] and some cancers [6, 7]. High dietary intake of carbohydrates is associated with low levels of total- and LDL cholesterol, as well as low concentrations of HDL-cholesterol and high levels of triglycerides [8]. On the other hand, consumption of low carbohydrate diets has been linked with elevated concentrations of inflammatory biomarkers [9–11], which could be underlying factors for cancer [12].

An inverse association was reported between adherence to LCD and risk of mortality from all causes, and also from cancer and cardiovascular disease [13–17]. Despite huge evidence on the protective role of low-carbohydrate diets in several cancers, some investigations have linked such diets with restricted tumor growth [16, 18]. Findings from other studies have provided null results with regards to the relation of LCD and incidence of cancers [19]. Data on the association between adherence to LCD and risk of breast cancer are scarce. In a published study from the Nurses’ Health Study, consumption of a low carbohydrate diet was associated with reduced risk of breast cancer in postmenopausal women who were estrogen receptor-negative [17].

The dietary intake of people in the Middle-East, due to its heterogeneity as well as other general characteristics, provides a unique opportunity to examine the relation between diet and disease. Most people in this region consume refined grains (refined bread and white rice) as their staple food [20]. In addition, due to the nutritional transition that is occurring in these countries, dietary fat intake, and in particular, detrimental fats, are increasing [21]. Such dietary characteristics make it reasonable to examine the contribution of different macronutrients to human health. Although the role of individual macronutrients in different cancers has been examined before [22, 23], no study is available linking breast cancer and a combination of macronutrients. Assessment of LCD in relation to chronic conditions is of great importance in Middle-Eastern countries since carbohydrates are the main source of energy in these countries and protein is expensive [24]. Dietary fat intake in this region is consumed at the level of DRI, however, type of dietary fat is a problem [25]. Given the aforementioned points, this study was done to assess the association between adherence to LCD and the odds of breast cancer in a case-control study in Iran.

## Subjects and methods

### Participants

We conducted a hospital-based, case-control study between 2014 and 2016 among Iranian women aged 19–80 years old. Cases (*n* = 486) were breast cancer patients that referred to surgery, chemotherapy or radiotherapy departments of Iran Cancer Institute that is located at a major teaching and general hospital, Imam Khomeini complex in Tehran. All patients had pathologically confirmed breast cancer within the past year. They had no history of any other cancers and long term dietary restrictions. Controls were 523 apparently healthy subjects [frequency-matched to cases by age (±10 years) and place of residence] admitted to the same hospital as healthy visitors, relatives and friends of non-cancer patients. They had no long term dietary restrictions. Individuals with a total energy intake of > 4500 or < 800 kcal/d (*n* = 116) as well as those who had no response to more than 70 items of FFQ (*n* = 25) were excluded from the study. Eventually, 412 cases and 456 controls remained for the current analysis. The study was approved by the Bioethics Committee of Iran Cancer Institute, Tehran University of Medical Sciences, Tehran, Iran, and all participants gave written informed consent at the beginning of the study.

### Assessment of dietary intake

Women with breast cancer were asked by trained interviewers to recall their usual diet over the preceding year using a validated semi-quantitative FFQ. It is clear that patients were likely to recall their dietary habits in the year prior to their diagnosis of breast cancer. The FFQ contained 168-food items with a standard serving size. Participants were asked to report their consumption on a daily, weekly or monthly basis. When the participants’ responses were not conformable with the given portion sizes, they were asked to report their own portion sizes for food items. Daily intake of reported food items was calculated and converted to grams per day using household measures. To compute energy and nutrient intakes, we used the USDA food composition database modified for Iranian foods [26, 27]. In a previous study, the validity of this FFQ was examined by comparing data from FFQ and the average of 12 dietary recalls [28]. The reliability was also examined through comparing data from two FFQs completed one year apart [29]. Findings from this validation study revealed that the FFQ provides reasonably valid and reliable data on long-term dietary intake [30].

### Construction of the low carbohydrate diet (LCD) score

The contribution of macronutrients to total energy intake was used to construct the LCD score. Initially, the percentages of energy intake from fats, proteins, and carbohydrates were computed. Then, individuals were divided into quintiles based on the percentages of each macronutrient. Subsequently, individuals in the highest quintile of fat and protein were given a score of 5 and those in the lowest quintile of these macronutrients were given a score of 1. Participants in the other quintiles of these macronutrients were given a corresponding score. Reverse scoring was used for carbohydrates; those with the highest intake were given a score of 1 and those with the lowest intake were given a score of 5. The scores were then summed up to compute the total low carbohydrate diet (LCD) score. The score for each participant ranged from 3 to 15. A higher score meant greater adherence to a low carbohydrate diet.

### Assessment of other variables

Weight was measured using digital scales to the nearest 100 g. Study participants were minimally clothed and without shoes during weighing. Height was measured while the women were standing and without shoes, using a tape meter. Body mass index (BMI) was calculated using measured weight and height. Data on physical activity was collected via a validated questionnaire, the Global Physical Activity Questionnaire (GPAQ) [31]. Patients were asked to recall their physical activity habits in the year preceding their cancer diagnosis. This questionnaire consists of 16 questions in 4 physical activity domains: job-related activities; transportation activities; recreation and sports activities; and sedentary behaviors. The measured data was processed according to the GPAQ Analysis Guide [32], and metabolic equivalent hours per week (MET-h/wk) values were calculated. Additional information on age, educational level, family history of breast cancer, alcohol and tobacco use, age at menarche, marital history, pregnancy history, parity, infertility treatment, age at menopause, postmenopausal hormone therapy, and contraceptive use were collected via questionnaire during a face-to-face interview.

### Statistical methods

Analyses were done in the total study population as well as stratified by pre- and postmenopausal status. Individuals were categorized based on quartiles of LCD scores. We used one-way ANOVA and chi-square tests to compare continuous and categorical variables, respectively, across quartiles of LCD score. Multivariable logistic regression models were used to assess the association between adherence to LCD and risk of breast cancer. The analyses were first adjusted for age (continuous) and additionally for physical activity (continuous), family history of breast cancer (yes vs. no), educational level (categorical), parity (nulliparous, 1, 2–3, ≥4), oral contraceptive use (yes vs. no), menopausal hormone use (yes vs. no), tobacco use (yes vs. no), alcohol use (yes vs. no), infertility treatment (yes vs. no), marital status (married, unmarried). We further controlled for dietary intake of vitamin B6, iron, folic acid, vitamin A and vitamin E (all continuous). Finally, we made adjustments for body mass index (continuous). The trend of odds ratios across quartiles of LCD score was examined by considering the median value of LCD score in each category as a continuous variable. *P* values < 0.05 were considered statistically significant. The analysis was performed by STATA version 14 (State Corp., College Station, TX).

## Results

Patients with BC were slightly older (46.3 νs. 44.2 years, *P* = 0.003), had lower BMI (28.1 νs. 28.8 kg/m^2^, *P* < 0.01), and were more likely to have a family history of breast cancer (10.1 νs. 1.54, *P* < 0.001) compared to controls (Table 1). They were less likely to be physically active (20.1 νs. 26.4 MET h/wk., *P* = 0.001), married (81.1 ν. 84.4%, *P* < 0.001), use oral contraceptives (52.8 vs. 61.3%, *P* = 0.02), use postmenopausal hormones (0.49 vs. 1.97%, *P* = 0.05) and drink alcohol (2.43 vs. 5.83%, *P* = 0.01) than controls (Table 1). Patients also had a lower intake of red meat and protein. The mean LCD score of participants was 8.9 ± 2.5 (8.9 ± 2.6 in cases and 9.0 ± 2.5 in controls).

**Table 1:** Baseline characteristics according to case-control

	case (*n* = 412)		control (*n* = 456)		*P*^a^
	Mean	SD	Mean	SD	
Median LCD score	8.9	2.6	9.0	2.6	0.27
Age, year	46.3	10.4	44.2	11.3	0.003
BMI (kg/m2)	28.1	5.2	28.8	6.1	0.03
Physical activity (MET h/wk)	20.1	25.1	26.4	37.1	0.001
Age at menarche, y	13.0	2.4	12.9	2.6	0.31
Educational level, n%					
Un university	83.2		84.3		0.66
University	16.7		15.7		
Married, %	81.1		84.4		< 0.001
Family history of breast cancer (yes), %	10.19		1.54		< 0.001
Oral contraceptive use (yes), %	52.8		61.3		0.02
Postmenopausal hormone use, %	0.49		1.97		0.05
Current smoking, %	3.4		4.9		0.21
Alcohol intake, %	2.43		5.83		0.01
Fertility treatment, %	4.6		6.4		0.26
Nulligravid, %	13.3		16.0		0.27
Parity					
Nulliparous/missing	43.4		42.9		0.94
1	8.7		9.2		
2–3	33.0		31.8		
≥ 4	14.8		16.0		
Whole grains (g/d)	93.2	96.4	91.2	101.2	0.38
Refined grains (g/d)	321.1	179.3	305.1	174.2	0.09
Fruits (g/d)	564.3	384.2	569.1	372.2	0.42
Vegetables (g/d)	301.6	242.1	316.6	211.5	0.16
Legumes (g/d)	47.3	51.8	51.8	81.5	0.16
Red meat (g/d)	13.8	18.9	16.2	21.5	0.04
Energy (kcal)	2572.1	820.2	2522.2	860.9	0.19
Carbohydrate intake (% of energy)	51.5	10.2	51.2	10.0	0.36
Protein intake(% of energy)	12.3	3.2	12.7	3.8	0.04
Fat intake (% of energy)	38.4	11.0	38.3	10.9	0.42
Fiber intake (g/d)	22.1	9.9	22.4	10.4	0.35

Values are mean (SD) or percentages

^a^χ^2^ Test for ordinal qualitative variables and ANOVA for continuous variables

Demographic, reproductive, and lifestyle characteristics of study participants (pre-and postmenopausal women) across quartile categories of LCD score are provided in Table 2. Mean (±SD) low-carbohydrate-diet score was 8.9 ± 2.5 for premenopausal and 9.0 ± 2.6 for postmenopausal women. Postmenopausal women in the top quartile of LCD score tended to be alcohol users, with a slightly lower age at menarche and lower family history of breast cancer compared to those in the bottom quartile.

**Table 2:** Baseline characteristics according to low-carbohydrate-diet score in participants

Characteristic	Premenopause (*n* = 567)								Postmenopause (*n* = 292)									
	Q1		Q2		Q3		Q4		*P*_trend_^a^	Q1		Q2		Q3		Q4		*P*_trend_^a^
	*n* = 172		*n* = 134		*n* = 174		*n* = 87			*n* = 88		*n* = 62		*n* = 90		*n* = 52		
	Mean	SD	Mean	SD	Mean	SD	Mean	SD		Mean	SD	Mean	SD	Mean	SD	Mean	SD	
Median score	5.8	1.0	8.43	0.49	10.5	0.49	12.7	0.83	< 0.001	5.6	1.0	8.53	0.50	10.5	0.50	12.63	0.79	< 0.001
Age, y	40.9	7.8	39.9	8.1	39.6	8.7	40.2	7.6	0.5	56.1	8.0	53.8	10.2	53.3	8.5	56.8	8.3	0.06
BMI (kg/m^2^)	27.7	5.1	27.9	5.3	28.1	6.7	27.6	4.4	0.85	29.4	6.1	29.6	6.0	29.3	5.2	29.7	5.8	0.96
Physical activity (MET h/wk)	24.8	31.3	19.8	25.6	25.0	30.6	25.4	35.9	0.4	23.2	22.1	24.7	34.1	23.6	47.4	21.8	29.1	0.97
Age at menarche, y	13.1	2.2	13.0	2.3	13.1	2.1	13.2	1.7	0.85	13.6	1.5	12.4	2.8	13.0	2.5	13.4	1.8	0.01
Educational level, n%																		
None	6.98		4.48		5.17		4.6		0.19	26.1		17.7		22.2		21.1		0.48
Primary	26.7		20.1		20.1		26.4			32.9		19.3		24.4		23.1		
Secondary school	49.4		51.4		60.9		45.9			29.5		50.0		42.2		44.2		
University	16.8		23.8		13.7		22.9			11.3		12.9		11.1		11.5		
Married, %	84.8		83.5		86.2		87.3		0.91	81.8		80.6		78.8		80.7		0.92
Family history of breast cancer (yes), %	5.23		7.46		3.45		5.75		0.47	7.95		12.9		2.2		3.8		0.05
Oral contraceptive use (yes), %	59.8		54.6		59.2		58.0		0.67	54.7		61.6		52.3		50.9		0.64
Postmenopausal hormone use, %	0		1.49		0.57		0		0.29	3.4		4.8		2.2		0		0.43
Current smoking, %	1.16		4.48		2.87		3.45		0.36	6.82		6.45		5.56		9.6		0.21
Alcohol intake, %	1.74		5.97		5.78		4.60		0.21	0		4.84		3.33		9.62		0.03
Fertility treatment, %	4.65		4.48		5.17		8.05		0.65	5.68		6.45		8.89		1.92		0.42
Nulligravid, %	15.12		20.15		16.67		13.79		0.57	9.09		8.06		10.00		5.77		0.84
Parity																		
Nulliparous/missing	44.1		40.3		38.5		42.5		0.81	43.1		48.3		44.4		46.1		0.89
1	8.14		14.9		11.49		13.7			5.68		4.8		3.3		1.9		
2–3	37.7		36.5		40.2		33.3			20.4		27.4		23.3		23.1		
≥ 4	9.88		8.21		9.7		10.3			30.6		19.3		28.8		28.8		

Values are mean (*SD*) or percentages

^a^χ^2^ Test for ordinal qualitative variables and *ANOVA* for continuous variables

Table 3 shows the mean daily dietary intake of food groups and nutrients across the quartiles of LCD score in pre-and postmenopausal women. Pre- and postmenopausal women in the top quartile of LCD score had a higher intake of red meat, full-fat dairy, protein, fat, saturated fat, and vitamin E and lower intake of whole grains, refined grains, fruits, and carbohydrate than those in the lowest quartile. In postmenopausal women, being in the top quartile of LCD score was associated with a lower intake of energy. Greater adherence to LCD in premenopausal women was associated with higher intake of vitamin A and lower intake of iron.

**Table 3:** Dietary intakes across categories of low carbohydrate score

variable	premenopause									postmenopause								
	Quartile1		Quartile2		Quartile3		Quartile4		*p*_trend_	Quartile1		Quartile2		Quartile3		Quartile4		*p*_trend_
N	n = 172		n = 134		n = 174		n = 87			n = 88		n = 62		n = 90		n = 52		
LCD score	3–7		8–9		10–11		12–15			3–7		8–9		10–11		12–14		
	Mean	SD	Mean	SD	Mean	SD	Mean	SD		Mean	SD	Mean	SD	Mean	SD	Mean	SD	
Food groups																		
Whole grains (g/d)	122	127	104	110	61	61	58	55	< 0.001	126	126	108	89	92	79	58	49	< 0.001
Refined grains (g/d)	408	213	339	182	287	135	227	118	< 0.001	359	180	291	175	263	119	185	103	< 0.001
Fruits (g/d)	643	410	528	343	524	389	500	319	0.004	660	450	621	364	552	342	467	274	0.01
Vegetables (g/d)	285	213	307	239	300	219	326	243	0.56	313	249	322	176	332	227	331	252	0.95
Legumes (g/d)	43	38	54	102	48	71	60	83	0.27	42	54	60	60	44	56	59	65	0.11
Red meat (g/d)	13	12	16	18	15	14	23	40	0.003	11	11	16	11	10	8	19	40	0.02
Low fat dairy (g/d)	63	116	53	117	55	123	98	191	0.06	50	89	38	67	98	180	75	122	0.01
High fat dairy (g/d)	315	238	364	256	368	253	529	357	< 0.001	306	235	351	225	411	266	461	366	0.005
Nutrients																		
Energy (kcal)	2557		2520		2704		2579		0.23	2321		2492		2612		2249		0.03
Carbohydrate intake (% of energy)	62	4	54	3	44	8	41	6	< 0.001	63	4	54	3	45	8	42	4	< 0.001
Protein intake(% of energy)	11.9	1.9	12.7	3.3	11.2	4.1	14.3	3.9	< 0.001	12.1	1.9	12.9	3.3	12.3	4.5	15.1	3.2	< 0.001
Fat intake (% of energy)	28.7	4.9	35.5	5.9	47.1	10.5	46.4	6.8	< 0.001	27.3	5.2	35.1	6.2	44.7	11.1	44.6	6.0	< 0.001
Saturated fat (g/d)	25	13	32	17	52	34	43	24	< 0.001	22	12	33	20	49	32	37	18	< 0.001
Fiber intake (g/d)	23	9	22	10.4	21	11.3	21	10	0.25	23	11	23	9	20	9	21	9	0.09
Iron (mg)	22	10	20	8	18	9	20	8	0.005	21	9	22	9	19	8	19	10	0.11
Folic acid (μg)	269	102	264	127	256	120	275	126	0.6	272	115	299	163	253	103	280	127	0.16
Vitamin B6 (mg)	1.7	0.67	1.7	0.72	1.6	0.79	1.8	0.87	0.24	1.7	0.76	1.7	0.61	1.7	0.72	1.7	0.74	0.86
Vitamin E (mg)	18	10	21.1	10.7	27	13	26.3	15.9	< 0.001	13.8	6.1	18.4	9.7	24	13	21	11.8	< 0.001
Vitamin A (μg)	840	452	924	595	850	529	1024	584	0.03	872	522	948	439	957	460	1035	660	0.33

*P*-values were determined by the *ANOVA* test

Multivariable-adjusted odds ratios and 95% CIs for breast cancer across categories of LCD score are provided in Table 4. Although no significant association was observed between adherence to LCD and odds of breast cancer in the whole study population, a trend toward significant positive association was seen between consumption of LCD and odds of breast cancer in postmenopausal women; after controlling for several potential confounders, individuals in the third quartile of LCD score were 1.94 times more likely to have breast cancer than those in the lowest quartile (95% CI: 1.00, 3.76). This association was strengthened after controlling for dietary variables (2.50; 1.18–5.32). Even after further adjustment for BMI, this association remained significant (2.64; 1.23–5.67). No such significant relationship was observed in premenopausal women, either before or after controlling for confounders.

**Table 4:** Risk for breast cancer according to quartiles of the low-carbohydrate-diet score with stratification by menopausal status

	OR (95% CI)				*P*_Trend_^a^
	Quartile 1	Quartile 2	Quartile 3	Quartile 4	
Total					
No.of cases/controls (412/456)	130/132	87/110	127/140	68/74	
Crude	1	0.80 (0.55–1.16)	0.92 (0.65–1.29)	0.93 (0.62–1.40)	0.81
Model 1	1	0.82 (0.57–1.21)	0.95 (0.67–1.35)	0.93 (0.61–1.41)	0.89
Model 2	1	0.79 (0.53–1.20)	1.05 (0.73–1.53)	0.97 (0.62–1.51)	0.79
Model 3	1	0.80 (0.53–1.22)	1.14 (0.76–1.71)	0.99 (0.62–1.58)	0.46
Model 4	1	0.84 (0.55–1.29)	1.22 (0.81–1.84)	1.00 (0.63–1.60)	0.37
Premenopause					
No.of cases/controls (267/300)	90/81	58/76	76/99	43/44	
Crude	1	0.68 (0.43–1.08)	0.69 (0.45–1.05)	0.87 (0.52–1.47)	0.28
Model 1	1	0.70 (0.44–1.11)	0.70 (0.45–1.07)	0.88 (0.52–1.49)	0.31
Model 2	1	0.64 (0.39–1.06)	0.77 (0.48–1.21)	0.91 (0.52–1.59)	0.55
Model 3	1	0.68 (0.40–1.14)	0.82 (0.50–1.36)	0.92 (0.51–1.66)	0.75
Model 4	1	0.72 (0.42–1.22)	0.89 (0.53–1.47)	0.93 (0.51–1.69)	0.88
Postmenopause					
No.of cases/controls (145/147)	40/49	29/33	51/38	25/27	
Crude	1	1.07 (0.56–2.06)	1.64 (0.91–2.97)	1.13 (0.57–2.25)	0.23
Model 1	1	1.13 (0.58–2.18)	1.78 (0.97–3.23)	1.13 (0.57–2.27)	0.18
Model 2	1	1.09 (0.52–2.31)	1.94 (1.00–3.76)	1.32 (0.61–2.84)	0.11
Model 3	1	1.18 (0.53–2.61)	2.50 (1.18–5.32)	1.46 (0.65–3.27)	0.06
Model 4	1	1.20 (0.54–2.66)	2.64 (1.23–5.67)	1.46 (0.65–3.28)	0.05

^a^Trend based on median values of each quartile

Model 1: Adjusted for age

Model 2: further adjusted for physical activity, family history of breast cancer, menopausal hormone use, education, parity, oral contraceptive use, cigar smoking, alcohol consumption, fertility treatment, marital status

Model 3: further adjusted for vitamin E, iron, vitamin B6, folic acid, vitamin A

Model 4: additional adjusted for *BMI*

## Discussion

In this case-control study, we found that adherence to a low-carbohydrate diet was not significantly associated with increased odds of breast cancer in the study population, a trend toward significant positive association was seen between consumption of LCD and odds of breast cancer in postmenopausal women. To our knowledge, this is the first study examining the relationship between low-carbohydrate diet score and risk of breast cancer in a Middle- Eastern country.

Earlier studies on the association between dietary carbohydrate intake and several outcomes have mostly examined total carbohydrate intake alone or in the context of low-carbohydrate, high-protein (LCHP) or low-carbohydrate, high-fat diets [2, 17, 23, 33–35]. The low-carbohydrate dietary pattern we focused on here, is usually characterized by low intake of carbohydrates and high intake of proteins and fats [36]. Therefore, this approach considers all macronutrients and can provide better insight into the link between macronutrient intake and risk of breast cancer compared to individual intake of these macronutrients. We found that adherence to a low carbohydrate diet was not significantly associated with increased risk of breast cancer in the study population. Two previous studies have reported association between adherence to LCD and risk of breast cancer [17, 33]. In a cohort study of Swedish women, adherence to LCHP was not associated with increased risk of respiratory tract cancer, even though a significant association was observed in men [33]. Another study was conducted among postmenopausal women of the Nurses’ Health Study and demonstrated an inverse association between consumption of a vegetable-based LCD and estrogen receptor-negative (ER-) breast cancer risk [17]. In addition to these observational studies, the effect of a low carbohydrate ketogenic diet on several cancers has been examined [37]. It has been shown that a low carbohydrate ketogenic diet had beneficial effects on cancer cells [38]. In addition to the carbohydrate content of the diet, other carbohydrate-related indices were also examined in relation to the risk of breast cancer. Farvid et al. within the framework of the Nurses’ Health Study population failed to find any significant association between dietary glycemic index (GI), glycemic load (GL) and insulin load during adolescence, and risk of breast cancer during adulthood [39]. Similarly, two prospective cohort studies on French and Canadian women, revealed no association between dietary GI, GL and carbohydrate intake with overall breast cancer risk [40, 41]. In contrast, in a case-control study on Mexican women, a positive association between carbohydrate intake and risk of breast cancer was found [42]. The quality and quantity of macronutrients in the diet might explain the null association we found. Low-carbohydrate diets have been reported to obtain less than 26% of their energy from carbohydrates [37], whereas the amount of energy from carbohydrates among those with the greatest adherence to the LCD in the current study was 41.5%. This might further help in understanding the difference in findings.

When we analyzed the results by menopausal status, a trend toward significant association was observed between adherence to LCD and risk of breast cancer among post-menopausal women. Despite lack of a significant association between dietary GI and risk of breast cancer in the population, Canadian researchers found a positive association between high GI diets and increased risk of breast cancer among postmenopausal women [41]. In a case-control study among premenopausal Mexican women [43], an increased risk of breast cancer with higher intake of carbohydrate was seen. The relationship between diet and risk of breast cancer has been different in pre- and postmenopausal women. It seems that the contribution of diet to the risk of breast cancer is strong in premenopausal women. However, we failed to find such an association in premenopausal women and further studies are needed to clarify the association between adherence to LCD and odds of breast cancer.

Although there are no clear mechanisms on the link between low carbohydrate dietary patterns and risk of breast cancer, some studies suggest some potential mechanisms. High carbohydrate intake was associated with elevated blood glucose and insulin levels, which can promote glucose intolerance, insulin resistance and hyperinsulinemia. Warburg et al. expressed that cancer cells depend on glucose as a fuel [44]. Insulin resistance results in decreased levels of insulin-like growth factor (IGF) binding proteins 1 and 2, therefore, the availability of IGF-Ι, which can in turn increase tumor cell proliferation, would increase [45, 46]. Elevated levels of insulin and IGF-Ι result in higher levels of free estrogen and androgen via inhibiting the hepatic synthesis of the sex hormone binding globulin [47, 48]. Therefore, adherence to a diet with a low carbohydrate content might suppress tumor cell proliferation and regulate apoptosis via cell signaling pathways, the PI3K/Akt/Mtor and RAS/RAF/MEK/ERK sequences, which are insulin or IGF-Ι-dependent [7, 46, 49]. The significant association between circulating levels of IGF-Ι and increased risk of breast cancer in premenopausal women has previously been shown [50].

The strengths of the current study include considering several potential confounders, recruiting participants from a referral hospital, in which subjects are from all across the country. Stratified analysis by menopausal status is a strength of this study. However, several limitations need to be considered. First, due to the case-control design of the study with its inherent recall and selection bias, one cannot infer causality. Second, as with all epidemiologic studies that apply FFQ, misclassification of participants in terms of dietary intake cannot be excluded. Third, prominence in the third quartile may be due to chance as the sample size is limited in each quartile. Therefore further studies with larger sample sizes are needed to confirm this finding. Fourth, we did not gather information on the hormone receptor status of participants. This may affect our findings.

## Conclusion

Based on the present findings, we found no evidence on association between consumption of a low carbohydrate diet (LCD), and odds of breast cancer risk in the study population; however, adherence to LCD might be associated with increased odds of breast cancer in postmenopausal women. Further research, especially cohort studies are needed to confirm these findings.
